# Intrathecal pump therapy: a retrospective cohort with over 1500 pump-years of follow-up

**DOI:** 10.1016/j.bas.2026.106261

**Published:** 2026-08-08

**Authors:** V. Matthijs, S. Vandamme, W. Nagels, O. De Coster, K. Hanssens, W. Maenhoudt, J. Van Lerbeirghe, O. Van Damme, D. Vanhauwaert

**Affiliations:** a Department of Neurosurgery, AZ Delta, Menen-Torhout, Roeselare, Belgium; b Department of Anaesthesiology & Pain Management Centre, AZ Delta, Menen-Torhout, Roeselare, Belgium

**Keywords:** Intrathecal pump therapy, Compounding, Device complications, Survival analysis

## Abstract

**Introduction:**

Intrathecal therapy (ITT) is an established treatment for severe spasticity and chronic pain syndromes. However, long-term device-related complications, such as infections and surgical revisions, remain a significant clinical concern.

**Research question:**

This study aims to quantify the incidence of these complications in a large retrospective cohort and evaluate the impact of compounded intrathecal medications on device longevity.

**Methods:**

We conducted a retrospective cohort study of patients undergoing intrathecal pump implantation or replacement (2003–2025). Complication burdens were calculated and expressed as incidence per 100 pump-years. To evaluate temporal risk, Kaplan-Meier time-to-event analyses were performed for both infections and revisions. The impact of compounded therapy versus monotherapy on revision-free survival was compared using the Log-Rank test, and pump exchange intervals were analysed via the Mann-Whitney *U* test.

**Results:**

The cohort included 132 patients (328 pumps), yielding a cumulative follow-up of 1551.6 pump-years. Nine infections occurred, corresponding to a low incidence of 0.58 per 100 pump-years. Forty-eight surgical revisions were performed, resulting in an incidence of 3.1 per 100 pump-years. The use of compounded medications was not associated with a statistically significant reduction in time to first or subsequent pump exchanges, and Log-Rank testing confirmed no significant difference in overall revision-free survival distributions between the monotherapy and compounding cohorts.

**Conclusion:**

With over 1500 cumulative pump-years of follow-up, ITT was associated with a low infection incidence and a moderate, time-dependent revision burden. The utilization of compounded intrathecal medications did not inherently compromise device survivability, providing empirical reassurance for individualized off-label therapy management.

## Highlights

• 1500 pump years: large cohort series for outcome of intrathecal therapy.
• Infection and revisions rates expressed per pump-year of therapy.
• Compounded intrathecal medication showed no negative impact on device longevity.

## 1 Introduction

Intrathecal therapy is an established treatment for intractable spasticity, dystonia, chronic cancer, and non-cancer pain (Nuru et al., 2022; Bonouvrié et al., 2022; Hoffmann et al., 2025). Approved by regulatory bodies for spasticity treatment since the early ‘90’s, intrathecal baclofen therapy has been widely applied for various neurological conditions causing spasticity, e.g. spinal cord injury, multiple sclerosis, cerebral palsy, and stroke (Nuru et al., 2022; Gahier et al., 2025). For chronic cancer pain the first use of intrathecal morphine was described in 1981 and its use expanded in the ‘90’s for non-cancer pain (Hoffmann et al., 2025). There are several types of non-cancer pain conditions reportedly treated with intrathecal therapy, e.g. persistent spinal pain syndrome type 1 & 2, complex region pain syndrome, phantom limb pain… (Hoffmann et al., 2025).

The advantage of direct drug delivery to the central nervous system significantly reduces the dosage and minimizes the systemic side effects, while maximizing the therapeutic effect (Nuru et al., 2022; Reis et al., 2019). The physiological principle of intrathecal drug delivery relies on the direct administration of active pharmacological agents into the cerebrospinal fluid (CSF) within the intrathecal space (Deer et al., 2024; Patel et al., 2025). By bypassing the blood-brain barrier and avoiding first-pass hepatic metabolism, this targeted approach achieves highly concentrated therapeutic drug levels at specific spinal cord receptors (e.g., $GABA_{B}$ receptors for baclofen or μ-opioid receptors for morphine) at a fraction of the equivalent systemic dose (Reis et al., 2019; Deer et al., 2024). Consequently, intrathecal therapy significantly minimizes systemic side effects—such as somnolence, profound hypotension, and cognitive impairment—commonly associated with high-dose oral or intravenous regimens, while maximizing localized therapeutic efficacy (Nuru et al., 2022; Reis et al., 2019; Patel et al., 2025).

In intrathecal therapy there are only three medications approved by the Food and Drug Administration (FDA), namely baclofen, morphine, and ziconotide (Hoffmann et al., 2025; Deer et al., 2024). However, for chronic pain there is an increasing use of off-label medications, mainly created by compounding diverse types of medications (Hoffmann et al., 2025; Deer et al., 2024; Patel et al., 2025). Compounding is the practice of combining multiple agents in one pump for intrathecal delivery. Current manufacturer registries indicate up to 83% of off-label use in chronic non-cancer pain (Hoffmann et al., 2025; Deer et al., 2024). However, these off-label uses have been discouraged by manufacturers, due to the possibility of pump failures, shorter pump-lifetime, and dosing errors (Deer et al., 2024).

Despite its established benefits, long-term use of implanted intrathecal systems is associated with potential complications, which impact patient outcomes and quality of life (Bonouvrié et al., 2022; Gahier et al., 2025). These complications can be divided into device-related, surgical, and pharmacological issues (Gahier et al., 2025). Among these, infections and mechanical issues requiring surgical revision are the most relevant (Bonouvrié et al., 2022; Reis et al., 2019). Incidences of serious complications necessitating re-intervention have been reported to vary significantly (Reis et al., 2019). Infections rates alone show ranges from 6.3% to 19.7% (Reis et al., 2019; Masrour et al., 2024; Jaffal et al., 2026). Catheter-related problems, e.g. kinking, occlusion, migration …, are also frequent causes for revision surgeries (Bonouvrié et al., 2022; Feller et al., 2021; Vanhauwaert et al., 2009).

Given the heterogeneity in reported complication rates and extended duration of intrathecal therapy, there is a continuous need for real-world data to quantify the incidence of complications and infections. Additionally, there is a lacking amount of evidence in the context of compounded medication and the effect on pump-exchange intervals. This study aims to report the incidence of infections and revisions and explore the effect of compounded intrathecal therapy on pump-exchange times in a large cohort of patients receiving intrathecal therapy. This cohort is reported in accordance with the STROBE-guidelines (von Elm et al., 2007).

## 2 Material and methods

### 2.1 Study design

We conducted a retrospective observational cohort study at AZ Delta, including data from its predecessor institutions. All patients who underwent primary implantation or replacement of an intrathecal pump device during the study period were eligible for inclusion (01/01/2003-31/12/2025). All included patients were treated exclusively with the SynchroMed II intrathecal pump system (Medtronic, Inc., Minneapolis, MN), ensuring device homogeneity across the cohort. Patients were identified through the hospital pharmacy database, operative records, and historical archives of the Department of Neurosurgery.

### 2.2 Data collection

Demographic, clinical, and surgical data were extracted from electronic and paper medical records. The following variables were collected: age, sex, indication for intrathecal therapy (e.g. congenital spasticity, acquired spasticity, persistent spinal pain syndrome type II), intrathecal medication regimen (monotherapy versus compounding), dates of pump implantation and replacement, pump serial number, occurrence of device-related infection and causative microorganism, and potential risk factors for infection, including smoking status, body mass index (BMI), diabetes mellitus, and colonization with methicillin-sensitive or methicillin-resistant *Staphylococcus aureus* (MSSA/MRSA).

All variables were entered into Microsoft Excel (Microsoft Corp., Redmond, WA, USA) and analysed using SPSS version 31 (IBM Corp., Armonk, NY, USA).

### 2.3 Outcomes

The primary goal was the incidence of device-related infections and surgical revisions, expressed per 100 pump-years of therapy. Infections were defined as clinically suspected or microbiologically confirmed infections involving the pump or catheter system requiring medical or surgical intervention. Surgical revisions were defined as unplanned surgical interventions related to pump or catheter dysfunction, excluding scheduled pump replacements and infection-related removal.

Secondary goal was to explore if there was a difference between pump replacement intervals compared between patients receiving intrathecal monotherapy versus compounding.

### 2.4 Statistical analysis

Total follow-up time was calculated for each pump from the date of implantation to pump replacement, removal, or last clinical follow-up and expressed as pump-years of therapy. Incidence rates were calculated by dividing the total number of events by the cumulative number of pump-years and expressed per 100 pump-years. Kaplan-Meier analysis was performed for both time to revision and time to infection. Time to event was calculated from the most recent pump-exchange to the event. Patients could have had multiple pump exchanges. To level device time, it was calculated starting from the most recent pump exchange to time of last follow-up or patient deceased. Due to the limited number of overall revision and infection events, multivariate Cox proportional hazards regression was statistically underpowered to identify independent risk factors without violating the events-per-variable (EPV) assumption. As a secondary analysis, pump replacement intervals were compared between patients receiving monotherapy and compounded therapy. Replacement intervals were expressed in months for the first, second, and subsequent pump exchanges. Data distribution was assessed using histograms, Q–Q plots, and the Kolmogorov–Smirnov and Shapiro–Wilk tests. As normality could not be assumed, comparisons between groups were performed using the Mann–Whitney *U* test, with alpha = 0.05. Kaplan-Meier analysis was performed for both the monotherapy and compounded therapy group for time to revision. Comparisons of survival distributions between subgroups (e.g., monotherapy versus compounded therapy) were performed using the Log-Rank (Mantel-Cox) test, with alpha = 0.05.

## 3 Results

### 3.1 Patient and pump characteristics

In total 132 patients are included in this cohort, combined for a total of 328 implanted pumps. There were 61 female patients (46.2%, 71 male), average overall age at first implantation was 47.1 years. Average age specified per indication was 34.5, 52.7, and 46.9 years for congenital spasticity, acquired spasticity, and chronic non-cancer pain, respectively. Median time of pump-therapy was 126.5 months (IQR 71-205.8) and there was a median of 2 pumps per patients (IQR 1.25-3). Indication for implantation of intrathecal pump therapy was spasticity in 84 patients (63.6%) and chronic non-cancer pain in 48 patients (36.3%). Spasticity is further subdivided into congenital (31/84, 36.9%) and acquired (63.1%), a comprehensive overview is given in Table 1. Most frequent medication was baclofen (73, 55.3%), followed by morphine-clonidine (16, 12.1%), and morphine (7, 5.3%). A comprehensive overview is given in Table 2. Median time between index pump and first exchange was 79 months (IQR 72-80.25). The time between first and second exchange had a median value of 77.5 months (IQR 66.5-79). Between second and third exchange the median value was 77 months (IQR 53-79).

**Table 1:** Overview of different indications for intrathecal therapy. PSPS: persistent spinal pain syndrome; CRPS: complex.

Indication	Number of patients
**Congenital**	31
hemiparesis/hemiplegia	2
paraparesis/paraplegia	8
tetraparesis/tetraplegia	21
**Acquired**	53
hemiparesis/hemiplegia	10
paraparesis/paraplegia	24
tetraparesis/tetraplegia	19
**Chronic non-cancer pain**	48
PSPS I	7
PSPS II	37
Phantom limb pain	3
CRPS	1

**Table 2:** Overview of different intrathecal medications.

Medication	Number of patients
**Monotherapy**	81 (61,4%)
Baclofen	73
Morphine	7
Catapressan	1
**Compounding**	51 (38,6%)
Baclofen Morphine	5
Baclofen Clonidine	1
Baclofen Marcaine	2
Morphine Clonidine	16
Morphine Midazolam	1
Morphine Marcaine	8
Baclofen Morphine Marcaine	9
Morphine Clonidine Sufentanyl	1
Morphine Clonidine Chirocaine	1
Morphine Sufentanyl Chirocaine	1
Morphine Clonidine Chirocaine	1
Morphine Clonidine Marcaine	4
Morphine Midazolam Marcaine	1
Morphine Clonidine Sufentanyl Chirocaine	1

Analysis of perioperative risk factors identified 18 patients with diabetes (13.6%), 10 patients were colonized with MRSA/MSSA and required preoperative decontamination schedules (7.6%); 4 missing data (3%), and 34 patients were active smokers (25.8%).

### 3.2 Infection and revision incidence

**Fig. 1:**
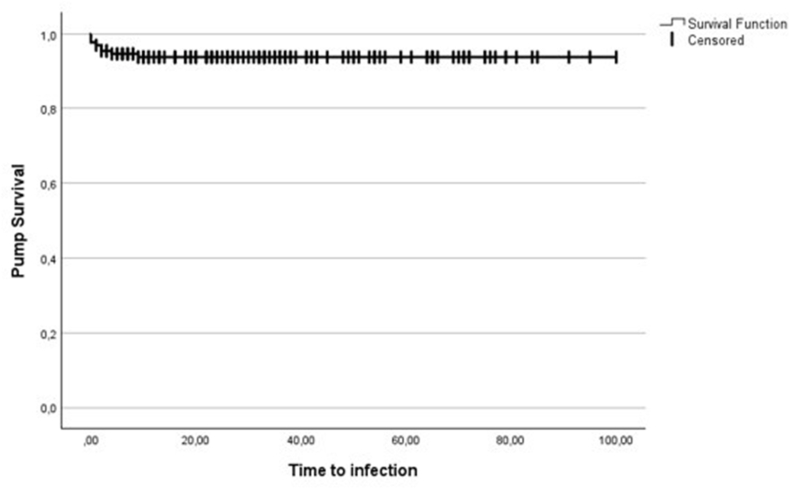
Kaplan-Meier curve showing infection free survival.

In total there were 9 infections (3%, 9/328 pumps), which required extraction of the system. The responsible agents were: *Staphylococcus aureus* (n = 3), *Staphylococcus epidermidis* (n = 1), *Streptococcus agalactiae* (n = 1), the other definitive diagnosis were lost between hospital file exchange (n = 4). The incidence for infection equals 0.58 infections per 100 pump-years of therapy (9/1551.6 pump-years). Time to infection analysis was made and visible in Fig. 1. Infection all occurred within the first 9 months after surgery, resulting in a one-year infection free survival of 93.8%, which remained stable at five-years.

**Fig. 2:**
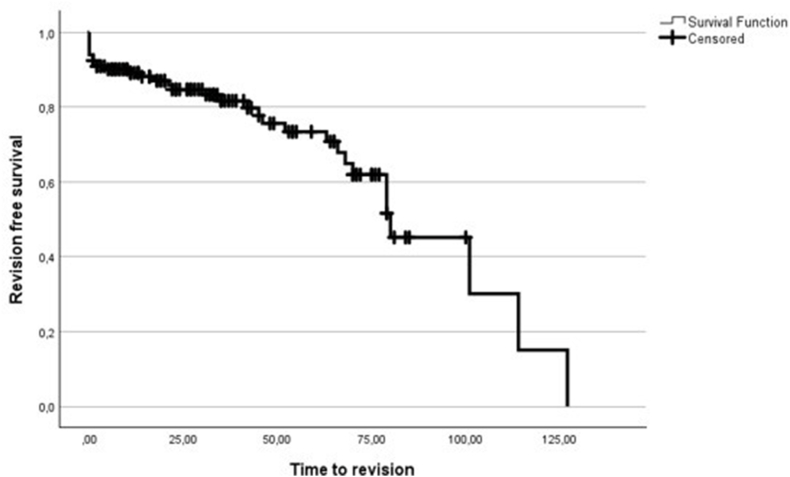
Kaplan-Meier curve showing revision free survival.

There were a total of 48 revisions (15%,48/328). Expressed as incidence, there were 3.1 revisions per 100 pump-years of therapy (48/1551.6 pump-years). 95 patients never required revision surgery (95/132, 72%). 24 patients required one revision (18.2%), 7 patients required two revisions (5.3%), 2 patients required three revisions (1.5%), 1 patient required 4 revisions (0.7%), and 3 patients had missing values. A comprehensive overview is given in Table 3. Time to revision analysis is shown in Fig. 2. This showed revision free survival at one-year of 89.1%, and five year survival of 73.4%. The median for revisions was reached at 80 months (6.6 years).

**Table 3:** Overview of the complications specified per category. Infections are specified per causative.

Complication	Number
Catheter related	27
dislocation	3
Dysfunction/occlusion	22
granuloma	2
**Pump related**	**17**
Pump dysfunction	15
Position/flip	2
**Varia**	**4**
Liquor leakage	1
Pocket hematom	3
**Infection**	**9**
*Staphylococcus aureus*	3
*Staphylococcus epidermidis*	1
*Streptococcus agalactiae*	1
Lost	4

### 3.3 Pump exchange intervals and compounded therapy

**Fig. 3:**
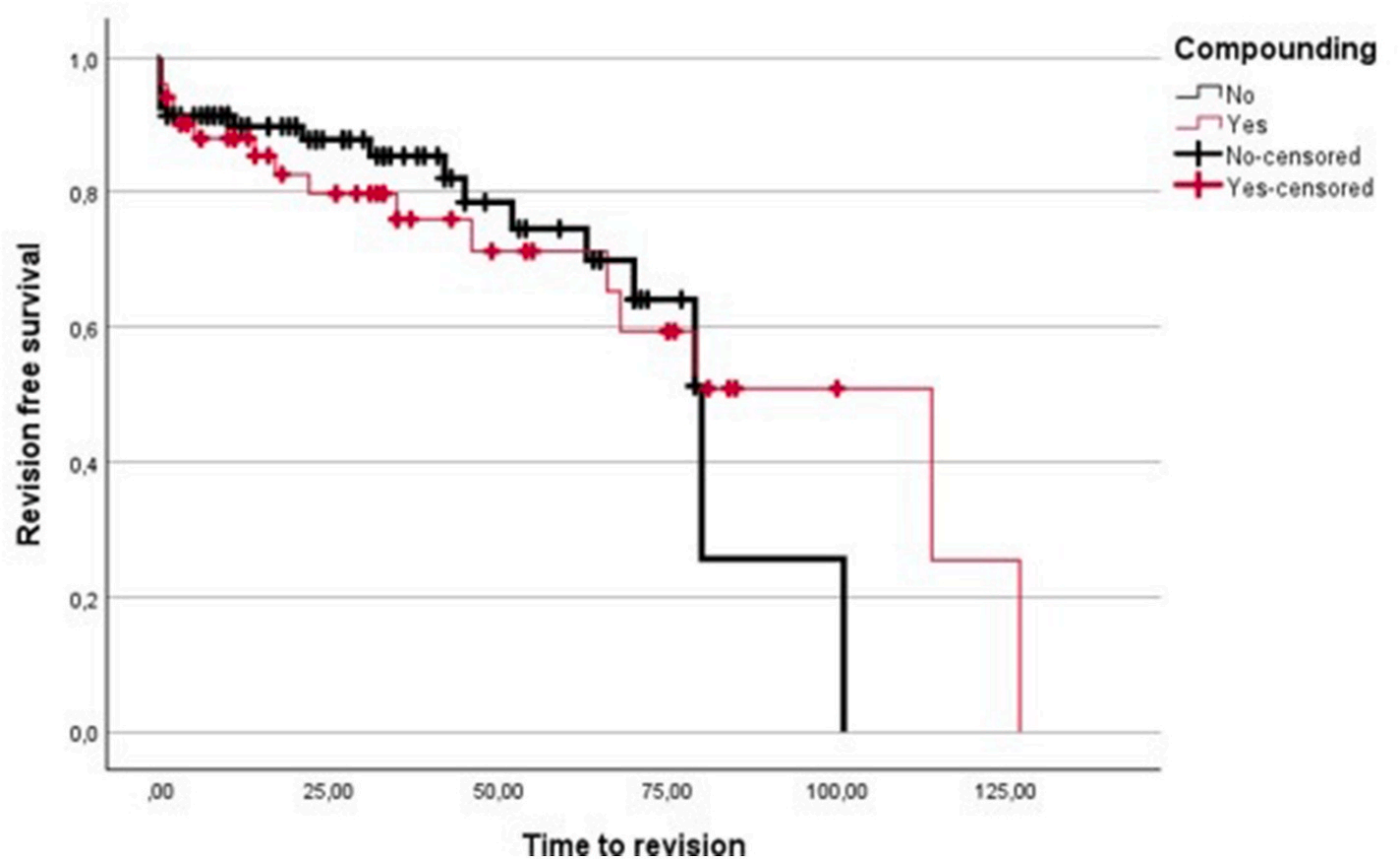
Kaplan-Meier curve showing revision free survival subdivided for compounding of medication.

81 patients received monotherapy, 51 compounded therapy. 53 patients had complete data for time between index pump and first exchange, and between first and second exchange (23 patients in monotherapy group, and 30 in the compounding group). There was no significant difference in distribution of median time between index and first pump exchange (p = 0.136). Equally, there was no significant difference in distribution of median time between first and second pump exchange (p = 0.135). Survival analysis was performed separately for monotherapy versus compounding, as seen in Fig. 3. Revision free survival rates were similar at one-year, respectively, 89.8% and 88.0%. Five-year survival was 74.3% in the monotherapy group, and 71.3% in the compounding group. Log-Rank test showed no statistically significant difference in survival distribution (p > 0.05).

## 4 Discussion

We report a retrospective cohort study with 1551.6 cumulative pump-year of therapy, which provides insights into incidence of complications and the effect of off-label therapy on pump-exchange intervals. The reported infections incidence is low, 0.58 per 100 pump-years, and we report a moderate surgical revision incidence of 3.1 per 100 pump years. Reporting incidences is methodically more robust than reporting prevalences or rates (Gahier et al., 2025). As incidences account for the cumulative exposure time and for the potential of multiple events per patient/pump, this is more representative of the true burden throughout the treatment (Gahier et al., 2025). This is pivotal in long-term therapies such as intrathecal therapy.

When comparing infections rates in the existing literature, there is considerable variability with studies reporting raw infections rates from 6.3% up to 15%, paediatric series reaching up to 44% (Nuru et al., 2022; Reis et al., 2019; Jaffal et al., 2026). The reported rate in this series is at the lower end of the reported literature. However, many of these series do no report incidences which makes more direct comparisons difficult. Gahier et al. adopted a similar approach and reports an overall complication incidence of 13 per 100 pump-years, extracting from their data an infection incidence of 1.5 per 100 pump years (Gahier et al., 2025). Our reported incidence of 0.58 is notably lower. Furthermore, our Kaplan-Meier time-to-event analysis adds temporal context not frequently detailed in similar cohorts. All infections occurred within the first 9 months post-implantation, resulting in a stable 93.8% survival plateau at five years. This confirms that infectious complications are almost exclusively early perioperative events. The identified pathogens, *Staphylococcus aureus* and *Staphylococcus epidermidis*, are consistent with the typical surgical site infections (Nuru et al., 2022).

Regarding surgical revisions, our incidence of 3.1 per 100 pump years implicates that both device related and catheter related complication are significant contributors for surgical re-interventions (Bonouvrié et al., 2022; Feller et al., 2021). Some studies report serious complication rates in up to 37% of patients, however these are often cumulative percentages and not incidences (Reis et al., 2019). Gahier et al. reported a total of 98 device related complications, which would correspond to an incidence of 6.2 per 100 pump-years (Gahier et al., 2025). This is in line with our findings that this long-term therapy is associated with a moderate revision burden. Our time-to-event analysis demonstrated a median revision-free survival of 80 months (6.6 years). This gradual, stepwise decline in survivability aligns tightly with the anticipated mechanical lifespan and battery depletion timelines of the device, rather than premature failures.

Our study also investigated the impact of compounding on device longevity. Off-label use is widely practiced in real clinical setting, with up to 83% of non-cancer pain patients receiving compounded medications (Hoffmann et al., 2025; Deer et al., 2024). Nevertheless, direct studies linking compounded mixtures to exchange intervals are scarce. Research has mainly focussed on the chemical stability and compatibility of various drugs or combinations of drugs within reservoirs, with some studies indication potential drug degradation of byproduct formation with negative effect on device longevity (Patel et al., 2025). Clinicians tailor compounded mixtures to achieve the best possible result for the individualized patient (Deer et al., 2024). Despite these theoretical risks our empirical survival analysis found no such penalty. Kaplan-Meier survival curves for revision free survival were not significantly different between the monotherapy and compounding group. Furthermore, our study found no statistically significant difference between compounding and reduction in time to first pump exchange (p = 0.136) or between subsequent pump exchanges (p = 0.135). This finding is highly clinically relevant, as it provides real-world reassurance to clinicians who tailor compounded mixtures to achieve optimal individualized patient analgesia (Deer et al., 2024; Patel et al., 2025).

This study has several limitations inherent to its retrospective design. First, while the cumulative follow-up of over 1500 pump-years is robust, the absolute number of events (9 infections, 48 revisions) restricted our statistical methodology. Specifically, the low event-per-variable ratio precluded the use of a multivariate Cox proportional hazards regression model. Consequently, while our univariate Kaplan-Meier analyses provide strong descriptive and comparative foundational data, we could not adjust for potential confounding clinical variables such as BMI or diabetes. Furthermore, while compounding did not broadly accelerate device failure in this cohort, the specific concentrations and specific combinations of the admixtures were not universally standardized. Therefore, larger, multi-center prospective registries are required to definitively rule out minor, combination-specific effects on catheter longevity.

## 5 Conclusion

This retrospective cohort study, with 1551.6 cumulative pump-years of therapy, provides valuable insights into the incidence of complications and the effect of off-label therapy on pump exchange intervals. The infection incidence was low at 0.58 per 100 pump-years, and the surgical revision incidence was moderate at 3.1 per 100 pump-years. Compounding did not significantly influence pump exchange intervals, with survival analysis confirming no statistically significant difference compared to monotherapy. These findings suggest that the clinical practice of compounding does not inherently compromise device survivability, offering empirical reassurance for individualized pain and spasticity management. Future prospective research is encouraged to validate these results and further investigate potential concentration-dependent effects on catheter longevity.

## Ethical approval

This study was approved by the local ethical committees (B117202500047) and was conducted in conjunction with the declaration of Helsinki.

## Authors' contributions

All authors contributed to the study conception and design. Material preparation, data collection and analysis were performed by Vincent Matthijs and Stijn Vandamme. The first draft of the manuscript was written by Vincent Matthijs, and all authors commented on previous versions of the manuscript. All authors read and approved of the final manuscript.

## Availability of data and materials

The datasets generated during and/or analysed during the current study are available from the corresponding author on reasonable request.

## Funding

No funding was received for this research.

## Declaration of competing interest

The authors declare that they have no known competing financial interests or personal relationships that could have appeared to influence the work reported in this paper.
